# Reduced Expression of the Polymeric Immunoglobulin Receptor in Pancreatic and Periampullary Adenocarcinoma Signifies Tumour Progression and Poor Prognosis

**DOI:** 10.1371/journal.pone.0112728

**Published:** 2014-11-14

**Authors:** Richard Fristedt, Jacob Elebro, Alexander Gaber, Liv Jonsson, Margareta Heby, Yulyana Yudina, Björn Nodin, Mathias Uhlén, Jakob Eberhard, Karin Jirström

**Affiliations:** 1 Department of Clinical Sciences Lund, Oncology and Pathology, Lund University, SE-221 85 Lund, Sweden; 2 Science for Life Laboratory, Royal Institute of Technology, 171 21 Stockholm, Sweden; 3 School of Biotechnology, AlbaNova University Center, Royal Institute of Technology, 106 91 Stockholm, Sweden; University of Pittsburgh, United States of America

## Abstract

The polymeric immunoglobulin receptor (pIgR) is a key component of the mucosal immune system that mediates epithelial transcytosis of immunoglobulins. High pIgR expression has been reported to correlate with a less aggressive tumour phenotype and an improved prognosis in several human cancer types. Here, we examined the expression and prognostic significance of pIgR in pancreatic and periampullary adenocarcinoma. The study cohort encompasses a consecutive series of 175 patients surgically treated with pancreaticoduodenectomy for pancreatic and periampullary adenocarcinoma in Malmö and Lund University Hospitals, Sweden, between 2001–2011. Tissue microarrays were constructed from primary tumours (n = 175) and paired lymph node metastases (n = 105). A multiplied score was calculated from the fraction and intensity of pIgR staining. Classification and regression tree analysis was used to select the prognostic cut-off. Unadjusted and adjusted hazard ratios (HR) for death and recurrence within 5 years were calculated. pIgR expression could be evaluated in 172/175 (98.3%) primary tumours and in 96/105 (91.4%) lymph node metastases. pIgR expression was significantly down-regulated in lymph node metastases as compared with primary tumours (p = 0.018). Low pIgR expression was significantly associated with poor differentiation grade (p<0.001), perineural growth (p = 0.027), lymphatic invasion (p = 0.016), vascular invasion (p = 0.033) and infiltration of the peripancreatic fat (p = 0.039). In the entire cohort, low pIgR expression was significantly associated with an impaired 5-year survival (HR = 2.99, 95% confidence interval (CI) 1.71–5.25) and early recurrence (HR = 2.89, 95% CI 1.67–4.98). This association remained significant for survival after adjustment for conventional clinicopathological factors, tumour origin and adjuvant treatment (HR = 1.98, 95% CI 1.10–3.57). These results demonstrate, for the first time, that high tumour-specific pIgR expression signifies a more favourable tumour phenotype and that low expression independently predicts a shorter survival in patients with pancreatic and periampullary cancer. The mechanistic basis for the putative tumour suppressing properties of pIgR in these cancers merits further study.

## Introduction

Adenocarcinomas arising in the pancreas and periampullary region are a heterogeneous group of neoplasms with the common feature of being highly aggressive and challenging to treat. Only 15–20% of the tumours are resectable at presentation [1], and there is an obvious lack of effective neoadjuvant-, adjuvant- and palliative radio-chemotherapeutic options, even with the advent of gemcitabine. Pancreatic cancer is the fourth most common cause of cancer related death, and the death rate has stayed stable over many years. The overall 5-year survival is 5%, all stages of the disease combined, and the median survival has been reported to be 5–8 months [2]–[4]. In resected periampullary carcinoma, morphological type seems to provide more important prognostic information than the tumour origin, with pancreatobiliary versus intestinal differentiation being associated with significantly shorter survival rates [5], [6]. Nevertheless, given the dismal prognosis for the group of pancreatic and periampullary carcinomas as a whole, the diagnostic and prognostic information provided by histopathological parameters is far from sufficient. Hence, there is a great need for additional molecular-based biomarkers, to better define clinically relevant subgroups of these tumours, and, thus, pave the way for novel treatment strategies.

The polymeric immunoglobulin receptor (pIgR) is a member of the immunoglobulin superfamily and it binds polymeric immunoglobulin molecules and presents them at the mucosal lining of the gastrointestinal tract and exocrine glands [7]. pIgR is a transmembrane protein that has three complementarity-determining regions (CDRs) on its extracellular part which form the ligand binding surface to which dimeric immunoglobulin A (IgA) is non-covalently attached [8]. pIgR binds IgA at the basolateral side of epithelial cells and the complex is then transcytosed across the cytoplasm to the apical part of the cell. The extracellular part of pIgR is then cleaved off as a secretory component (SC) bound to polymeric IgA protecting it from proteolytic degradation [8]. Hence, pIgR plays an important role in linking innate and adaptive immune responses. The extracellular component of pIgR can also be cleaved off to produce SC without being bound to IgA molecules and then acts as a scavenger on the mucosal lining [8]. A number of cytokines are known to regulate pIgR expression; interferon (IFN)-y (type 1 helper-T cells), tumour necrosis factor (TNF), interleukin-1 (IL-1), IL-4 (type 2 helper-T cells) [9].

Using the Human Protein Atlas as a tool for antibody based biomarker discovery [10], we recently identified pIgR as being differentially expressed in several major forms of cancer, whilst the expression in several types of normal tissues in general appears to be high (www.proteinatlas.org). Comparatively few studies have investigated the expression and prognostic significance of pIgR in human cancer, but the majority indicate associations of a high pIgR expression with a more favourable phenotype and an improved survival [11]–[17]. To date, we are only aware of one study reporting adverse prognostic implications of pIgR expression in human cancer, namely in hepatitis B-derived hepatocellular carcinoma, where high pIgR expression was found to be associated with a greater metastatic potential and poor prognosis [18].

pIgR has recently been demonstrated to be upregulated in pancreatic cancer cells upon exposure to stromal cells in vitro [19], but to the best of our knowledge, the expression and prognostic significance of pIgR has not yet been described in pancreatic cancer. The aim of this study was therefore to examine the expression of pIgR, and its prognostic impact, in primary tumours and paired lymph node metastases from a consecutive cohort of patients surgically treated with pancreaticoduodenectomy (PD) for pancreatic and periampullary adenocarcinoma (n = 175).

## Methods

### Patients

The study cohort is a previously described [20] retrospective consecutive series of 175 PD-specimens with primary adenocarcinomas surgically treated at the University hospitals of Lund and Malmö, Sweden, from January 1 2001 until December 31 2011. Data on survival were gathered from the Swedish National Civil Register. Follow-up started at the date of surgery and ended at death or at December 31 2013, whichever came first.

All haematoxylin & eosin stained slides from all cases were re-evaluated by one pathologist (JEL), blinded to the original report and outcome, with the decision on tumour origin being based on several criteria, as previously described [20].

Information on neoadjuvant and adjuvant treatment and recurrence, stratified for tumours of intestinal and pancreaticobiliary subtype, respectively, is summarized in Table 1.

**Table 1:** Adjuvant and neoadjuvant treatment, vital status and recurrence rate by morphological type.

	Intestinal type (n = 65)	Pancreatobiliary type (n = 110)
	n (% column)	n (% column)
**Adjuvant chemotherapy**		
No	47 (72.3)	51 (46.4)
5-FU analogue	5 (7.7)	8 (7.3)
Gemcitabine	7 (10.8)	45 (40.9)*
Gemcitabine + capecitabine	1 (1.5)	3 (2.7) **
Oxaliplatin + 5-FU analogue	4 (6.2)	1 (0.9)
Gemcitabine + oxaliplatin	1 (1.5)	2 (1.8)
**Five-year survival (months)**		
Mean	37.53	27.27
Median	38.40	25.26
Range	0.79–60.00	5.42–60.0
**Vital status at end of follow-up**		
Alive	31 (47.7)	26 (23.6)
Dead	34 (52.3)	84 (76.4)
**Recurrence**		
No	35 (53.8)	19 (17.4)
Yes	30 (46.2)	90 (82.6)
Local	4 (6.2)	30 (27.5)
Generalised	16 (24.6)	28 (25.7)
Liver	6 (9.2)	20 (18.3)
Lungs	4 (6.2)	12 (11.0)
*Missing*		*1*

* One patient received neoadjuvant treatment with radiotherapy + capecitabine.

**One patient received neoadjuvant therapy with radiotherapy + gemcitabine + capecitabine.

### Ethics Statement

All EU and national regulations and requirements for handling human samples have been fully complied with during the conduct of this project; i.e. decision no. 1110/94/EC of the European Parliament and of the Council (OJL126 18,5,94), the Helsinki Declaration on ethical principles for medical research involving human subjects, and the EU Council Convention on human rights and Biomedicine. The study was approved of by the Ethics committee of Lund University (ref nr 445/07), whereby the committee waived the need for consent other than by the option to opt out.

### Tissue microarray construction

Tissue microarrays (TMAs) were constructed using a semi-automated arraying device (TMArrayer, Pathology Devices, Westminister, MD, USA). A standard set of three tissue cores (1 mm) were obtained from each of the 175 primary tumours and from paired lymph node metastases in 105 cases, whereby one-three lymph node metastases were sampled in each case. In addition, adjacent benign-appearing pancreatic tissue was sampled from 50 cases using two (1 mm) tissue cores.

### Immunohistochemistry and staining evaluation

For immunohistochemical analysis of pIgR expression, 4 µm TMA-sections were automatically pre-treated using the PT Link system and then stained in an Autostainer Plus (DAKO; Glostrup, Copenhagen, Denmark) with a polyclonal, monospecific antibody; HPA012012, Atlas Antibodies AB, diluted 1∶200. The specificity of the antibody was confirmed by immunofluorescence, Western blotting and protein arrays (www.proteinatlas.org).

pIgR was exclusively expressed in the cytoplasm and cell membrane, in line with previous observations [16], [17]. The staining was annotated by two observers (RF, AG) whereby consensus for each core was reached in estimated fraction 0.0–1.0 (1 = 100%) of stained cells, while staining intensity was annotated as 0 = negative, 1 =  weak, 2 = moderate and 3 = strong. A multiplier of intensity (0–3) and fraction (0.0–1.0) for each core was calculated and a mean value of all annotated cores was used in the analyses.

### RNA Extraction and Quantification of *PIGR* Gene Expression with RT-qPCR

Total RNA was extracted from paraffin-embedded tissue sections using AllPrep DNA/RNA FFPE extraction kit (QIAGEN, Germantown, MD, USA), according to manufacturer's instructions. Total RNA (1 µg) was reverse transcribed to cDNA using an SuperScript VILO cDNA Synthesis Kit (Life Technologies, Waltham, MA USA) 50 ng of cDNA was mixed with 0.5 µM KiCqStart SYBRGreen predesigned primers (Sigma-Aldrich, St. Louis, MO USA) and SYBR Select Master mix (Life Technologies, Waltham, MA USA) and amplified in a StepOne Real-Time PCR Systems (Applied Biosystems, Foster City, CA USA) with standard cycling parameters. The samples were analysed and normalised against a housekeeping gene (GAPDH) using the StepOne software (Applied Biosystems, Foster City, CA).

### Statistical analysis

Non-parametric Wilcoxon-Rank, Mann-Whitney U and Kruskal-Wallis tests were applied for analyses of differences in the distribution of pIgR expression in primary tumours and lymph node metastases, and according to clinicopathological characteristics. Two patients who had received neoadjuvant chemotherapy were excluded from the correlation analyses and survival analyses and three additional patients were excluded from the survival analyses; two who died within one month from surgery due to complications and one who emigrated 5 months after surgery.

Classification and regression tree (CRT) analysis [21] was used to assess optimal prognostic cut off for pIgR expression in relation to 5-year overall survival (OS) and recurrence free survival (RFS). RFS was defined from the date of surgery to the date of locoregional or distant recurrence.

Kaplan Meier analysis and the log rank test were applied to estimate differences in 5-year OS and RFS in strata according to high and low pIgR expression. Hazard ratios (HR) and confidence intervals (CI) at the 95% level for death and recurrence within 5 years were calculated by Cox regression proportional hazard's modelling in both unadjusted analysis and in a multivariable model adjusted for age, sex, tumour (T-) stage, nodal (N-) stage, differentiation grade, lymphatic invasion, vascular invasion, perineural invasion, infiltration in peripancreatic fat, resection margins, tumour location, and adjuvant chemotherapy. A backward conditional method was used for variable selection in the adjusted model. All tests were two sided. P-values <0.05 were considered significant. All statistical analyses were performed using IBM SPSS Statistics version 22.0 (SPSS Inc., Chicago, IL, USA).

## Results

### pIgR expression in primary tumours and paired metastases

**Figure 1:**
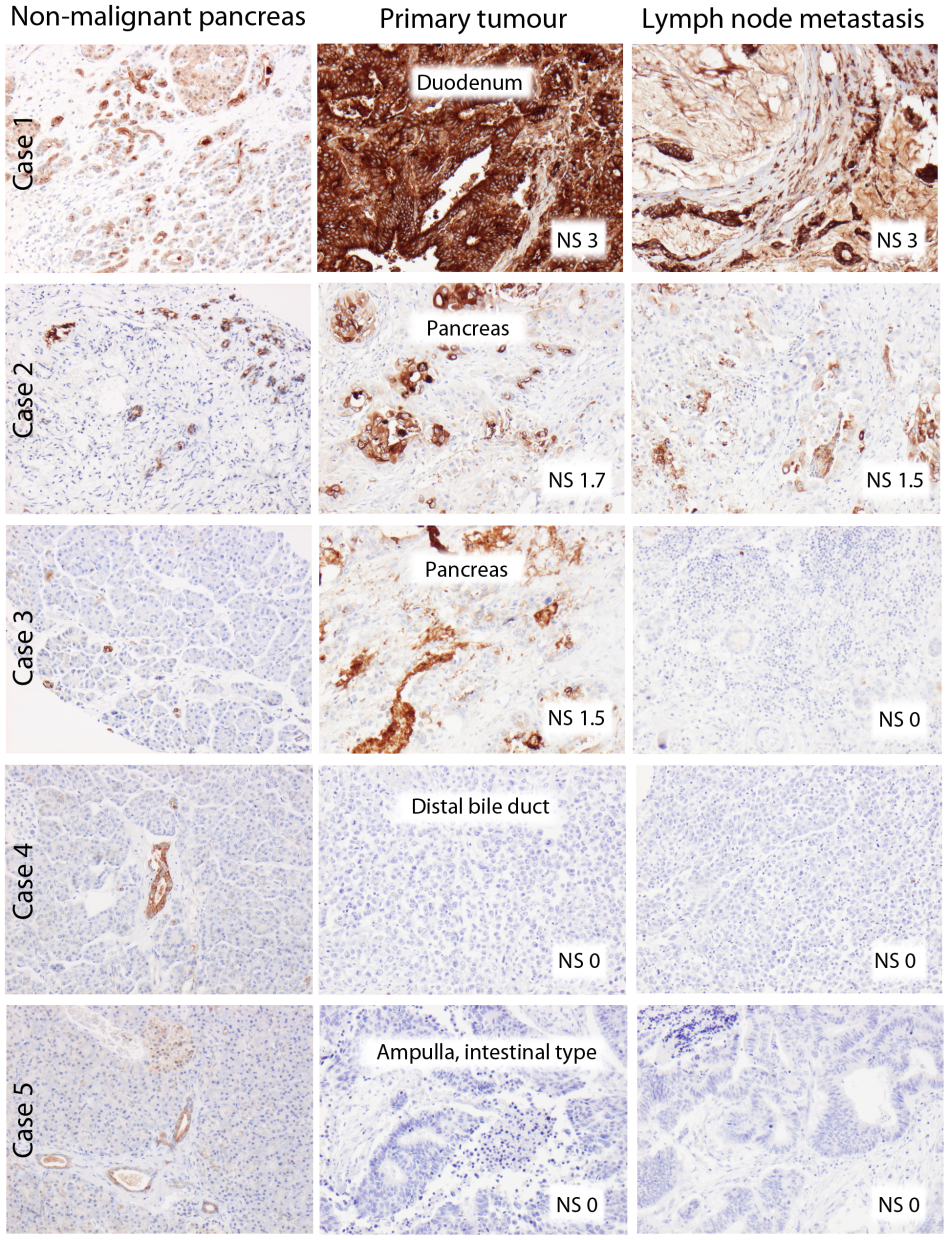
Sample immunohistochemical images. pIgR expression in non-malignant pancreatic tissue primary tumour and paired lymph node metastasis (right column) from five cases of different origins and with different nuclear scores (NS).

pIgR expression could be evaluated in all 50 samples with non-malignant tissue, in 172/175 (98.3%) of the primary tumours and in 96/105 (89.5%) of the sampled lymph node metastases. In benign-appearing pancreatic tissue, moderate to high expression of pIgR was denoted in the ducts, whereas the acini were negative. A total number of 25 (14.5%) primary tumours and 20 (20.8%) metastases were completely negative for pIgR expression, and in the other cases, pIgR was expressed in varying fractions and intensities. Sample IHC images from five different cases are shown in Figure 1. In two of these cases, PIGR mRNA expression levels were successfully evaluated in formalin-fixed paraffin-embedded tissue from paired primary tumours and lymph node metastases. The results demonstrated a good correlation between protein and gene expression and the downregulated protein expression from primary tumour to metastasis in one case was also confirmed at the mRNA level (Figure S1).

**Figure 2:**
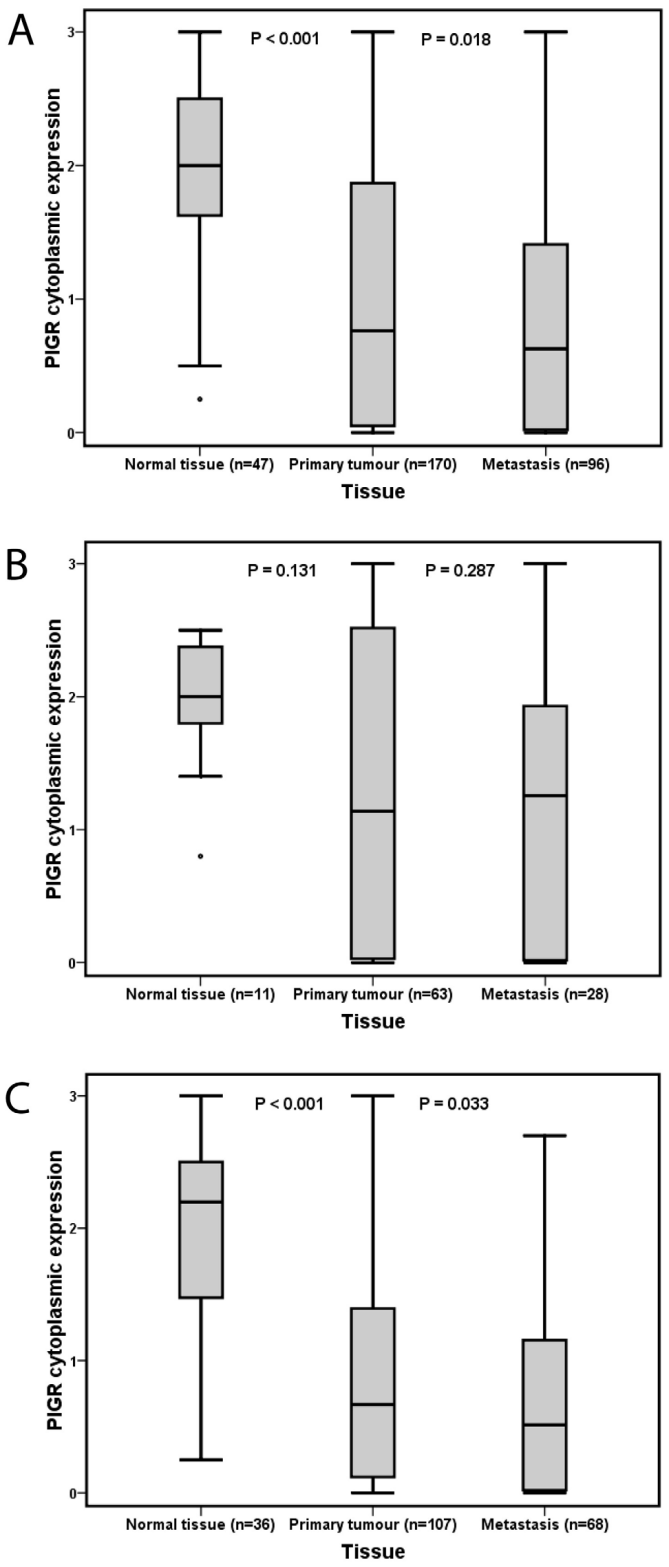
pIgR expression in primary tumours and metastases. Box plots visualizing the distribution of pIgR expression (total score) in primary tumours and lymph node metastases in (A) the entire cohort, (B) tumours with intestinal type morphology and (C) tumours with pancreatobiliary type morphology.

As demonstrated in Figure 2, pIgR expression was significantly higher in benign pancreatic tissue compared to primary and metastatic tumours in the full cohort as well as in separate analysis of intestinal and pancreatobiliary type tumours (p<0.001 for all, Figure 2 A–C). pIgR expression was significantly lower in lymph node metastases compared to primary tumours in the entire cohort (p = 0.018, Figure 2A), and in pancreatobiliary type (p = 0.033, Figure 2C) but not in intestinal type (Figure 2B) tumours.

### Associations of pIgR expression with clinicopathological characteristics

As demonstrated in Table 2, pIgR expression was significantly associated with tumour origin (p = 0.033), with the highest expression in tumours of duodenal origin, and the lowest expression in tumours of pancreatic origin. There were also significant inverse associations between pIgR expression and perineural invasion (p = 0.027), tumour differentiation (p<0.001) and lymphatic (p = 0.016), vascular (p = 0.033) and peripancreatic fat growth (p = 0.039). There was a trend, however non-significant, towards an association with resection margins (p = 0.067). There was no significant correlation between pIgR expression and age, gender, T-stage, N-stage or tumour size.

**Table 2:** Associations of pIgR expression in primary tumours with clinicopathological parameters.

Factor*	pIgR expression	
	median (range)	*p-value*
**Age**		
Q1 (n = 39)	0.95 (0.00–3.00)	0.870
Q2 (n = 45)	0.57 (0.00–3.00)	
Q3 (n = 43)	0.76 (0.00–3.00)	
Q4 (n = 42)	0.97 (0.00–3.00)	
**Gender**		
Female (n = 84)	0.68 (0.00–3.00)	0.612
Male (n = 88)	0.80 (0.00–3.00)	
**Tumour origin**		
Duodenum (n = 14)	2.60 (0.00–3.00)	0.033
Papilla-ampulla intestinal type (n = 49)	0.87 (0.00–3.00)	
Papilla-ampulla pancreatobiliary type (n = 19)	1.12 (0.00–2.48)	
Distal bile duct (n = 45)	0.62 (0.00–3.00)	
Pancreas (n = 45)	0.51 (0.00–2.75)	
**T-stage**		
T1 (n = 6)	1.16 (0.08–2.78)	0.410
T2 (n = 23)	1.28 (0.00–3.00)	
T3 (n = 103)	0.72 (0.00–3.00)	
T4 (n = 40)	0.81 (0.00–3.00)	
**N-stage**		
N0 (n = 65)	0.80 (0.00–3.00)	0.206
N1 (metastasis in 1-3 lgl, n = 64)	0.89 (0.00–3.00)	
N2 (metastasis in 4 or more lgl, n = 43)	0.49 (0.00–3.00)	
**Differentiation grade**		
Well (n = 11)	2.07 (0.08–3.00)	<0.001
Moderate (n = 61)	1.28 (0.00–3.00)	
Poor (n = 96)	0.46 (0.00–3.00)	
**Tumour size**		0.655
< = 20 mm (n = 38)	0.62 (0.00–3.00)	
>20 mm (n = 134)	0.80 (0.00–3.00)	
**Resection margins**		
R0 (n = 23)	1.27 (0.00–2,90)	0.067
R1 (n = 94)	0.59 (0.00–3.00)	
RX (n = 55)	1.06 (0.00–3.00)	
**Perineural growth**		
Absent (n = 68)	1.20 (0.00–3.00)	0.027
Present (n = 104)	0.64 (0.00–3.00)	
**Lymphatic growth**		
Absent (n = 63)	1.12 (0.00–3.00)	0.016
Present (n = 109)	0.62 (0.00–3.00)	
**Vascular growth**		
Absent (n = 130)	0.89 (0.00–3.00)	0.033
Present (n = 42)	0.29 (0.00–2.95)	
**Peripancreatic fat growth**		
Absent (n = 65)	1.12 (0.00–3.00)	0.039
Present (n = 107)	0.67 (0.00–3.00)	

Age quartiles: Q1 = 38–61, Q2 = 62–67, Q3 = 68–72, Q4 = 73–84.

T-Stage = Tumour stage.

N-Stage = Nodal stage.

Resection margin: R0 = free resection margin, R1 = microscopic tumour invasion within 1 mm of the medial margin, RX = not assessable microscopic invasion.

### Associations of pIgR expression with survival

**Figure 3:**
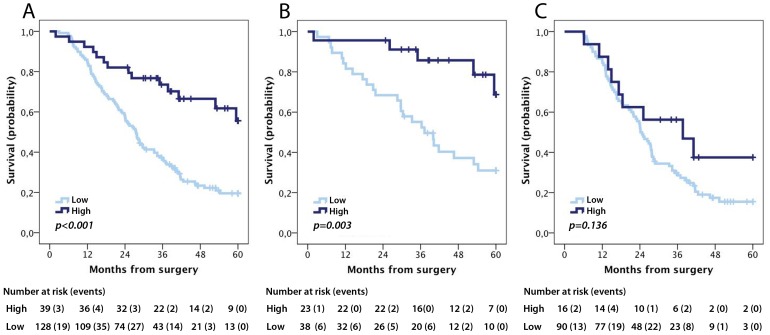
Kaplan-Meier estimates of 5-year overall survival according to pIgR expression. Five-year overall survival according to high and low pIgR expression in (A) the entire cohort, (B) intestinal type tumours and (C) pancreatobiliary type tumours.

**Figure 4:**
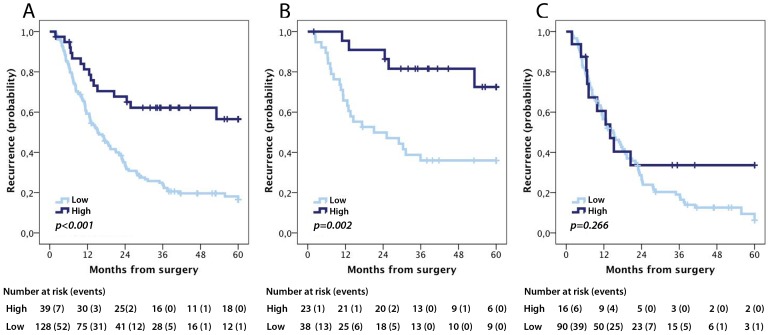
Kaplan-Meier estimates of recurrence free survival according to pIgR expression. Recurrence-free survival according to high and low pIgR expression in (A) the entire cohort, (B) intestinal type tumours and (C) pancreatobiliary type tumours.

According to the CRT analysis a prognostic cut-off of 1.892 was adopted (Figure S2). Kaplan-Meier analysis revealed a significantly reduced 5-year OS for patients with tumours displaying low pIgR expression (logrank p<0.001, Figure 3A). In subgroup analysis by morphological type, this association remained significant for intestinal type (duodenum and ampullary intestinal type) tumours (logrank p = 0.003, Figure 3B), but not in pancreatobiliary type (ampulla-pancreatobiliary type, distal bile duct and pancreatic) tumours (logrank p =  0.136, Figure 3C). Similar trends were seen for RFS (Figure 4 A–C).

The associations of pIgR expression were confirmed in unadjusted Cox regression analysis for both 5-year OS (HR 2.99, 95% CI 1.71–5.25 for the entire cohort and 3.90, 95% CI 1.49–10.21 for intestinal type tumours, Table 3) and RFS (HR 2.89, 95% CI 1.67–4.98 for the entire cohort and HR 4.01, 95% CI 1.53–10.54 for intestinal type tumours, Table 4). In adjusted analysis, pIgR remained an independent prognostic factor for 5-year OS (Table 3) in the entire cohort (HR 1.98, CI 95% 1.10–3.57) and in intestinal type tumours (HR 3.76 CI 95% 1.27–11.11). pIgR expression did not remain an independent predictor of RFS in the adjusted model (Table 4).

**Table 3:** Unadjusted and adjusted hazard ratios for death within five years in the entire cohort, intestinal and pancreatobiliary type tumours.

	Entire cohort			Intestinal type			Pancreatobiliary type		
		Unadjusted	Adjusted		Unadjusted	Adjusted		Unadjusted	Adjusted
	n(events)	HR(95%CI)	HR(95%CI)	n(events)	HR(95%CI)	HR(95%CI)	n(events)	HR(95%CI)	HR(95%CI)
**Age**									
Continuous	167 (112)	1.01 (0.98–1.03)	1.03 (1.00–1.05)	61 (30)	1.02 (0.98–1.06)	1.05 (1.01–1.10)	106 (82)	0.99 (0.96–1.02)	1.01 (0.98–1.05)
**Gender**									
Female	84 (49)	1.00	1.00	34 (13)	1.00	1.00	50 (36)	1.00	1.00
Male	83 (63)	1.41 (0.97–2.05)	1.15 (0.77–1.72)	27 (17)	1.76 (0.87–3.57)	1.54 (0.63–3.78)	56 (46)	1.20 (0.78–1.86)	1.19 (0.74– 1.91)
**Tumour origin**									
Duodenum	13 (5)	1.00	1.00	13 (5)	1.00	1.00			
Ampulla-Intestinal type	48 (25)	1.50 (0.57–3.91)	1.41 (0.47–4.22)	48 (25)	1.58 (0.61–4.09)	1.95 (0.38–9.94)			
Ampulla-Pancreatobiliary type	19 (16)	4.08 (1.48–11.20)	1.11 (0.33–3.67)				19 (16)	1.00	1.00
Distal Bile duct	44 (32)	2.94 (1.14–7.57)	1.97 (0.58–6.74)				44 (32)	0.73 (0.40–1.34)	1.09 (0.54–2.21)
Pancreas	43 (34)	3.63 (1.41–9.31)	2.35 (0.66–8.33)				43 (34)	0.91 (0.50–1.65)	1.09 (0.55–2.16)
**Tumour size**									
Continuous	167 (112)	1.02 (1.00–1.03)	1.00 (0.98–1.03)	61 (30)	1.00 (0.97–1.02)	1.03 (0.99–1.08)	106 (82)	1.03 (1.01–1.05)	1.01 (0.98–1.04)
**T-stage**									
T1	6 (3)	1.00	1.00	4 (2)	1.00	1.00	2 (1)	1.00	1.00
T2	20 (9)	1.03 (0.28–3.80)	0.86 (0.22–3.35)	10(3)	0.56 (0.12–2-49)	0.83 (0.12–6.02)	10 (6)	1.43 (0.17–11.85)	0.80 (0.08–7.78)
T3	102 (69)	2.44 (0.77–7.76)	1.12 (0.32–3.84)	25 (9)	0.63 (0.17–2.34)	1.31 (0.18–9.30)	77 (60)	2.95 (0.41–21.34)	0.85 (0.10–7.08)
T4	39 (31)	2.98 (0.91–9.77)	1.05 (0.29–3.81)	22 (16)	1.67 (0.48–5.78)	2.27 (0.26–19.82)	17 (15)	3.77 (0.49–28.71)	2.19 (0.10–49.23)
**N-stage**									
N0	62 (33)	1.00	1.00	33 (15)	1.00	1.00	29 (18)	1.00	1.00
N1 (metastasis in 1-3 lgl)	64 (46)	2.08 (1.33–3.27)	1.83 (1.12–2.96)	19 (9)	1.05 (0.47–2.35)	0.61 (0.22–1.73)	45 (37)	2.40 (1.35–4.29)	2.85 (1.57–5.17)
N2 (metastasis in 4 or more lgl)	41 (33)	2.72 (1.66–4.45)	1.68 (0.96–2.94)	9 (6)	1.83 (0.72–4.66)	2.78 (0.91–8.52)	32 (27)	2.59 (1.40–4.78)	2.45 (1.30–4.63)
**Differentiation grade**									
Well-moderate	69 (36)	1.00	1.00	30 (12)	1.00	1.00	39 (24)	1.00	1.00
Poor	98 (76)	2.42 (1.62–3.60)	1.84 (1.21–2.81)	31 (18)	1.88 (0.92–3.82)	1.55 (0.60–3.98)	67 (58)	2.44 (1.50–3.95)	2.13 (1.28–3.54)
**Involved margins, status**									
R0	23 (6)	1.00	1.00	17 (4)	1.00	1.00	6 (2)	1.00	1.00
R1 & Rx	144 (106)	3.83 (1.68–8.73)	2.12 (0.91–4.98)	44 (26)	2.11 (0.82–5.50)	1.89 (0.68–5.27)	100 (80)	3.49 (0.86–14.24)	2.57 (0.62–10.60)
**Lymphatic growth**									
Absent	60 (29)	1.00	1.00	28 (7)	1.00	1.00	32 (22)	1.00	1.00
Present	107 (83)	2.20 (1.44–3.37)	1.11 (0.70–1.77)	33 (23)	3.82 (1.65–8.86)	8.70 (2.89–26.18)	74 (60)	1.51 (0.92–2.48)	0.95 (0.54–1.68)
**Vascular growth**									
Absent	126 (72)	1.00	1.00	56 (25)	1.00	1.00	70 (47)	1.00	1.00
Present	41 (40)	3.46 (2.32–5.15)	3.01 (1.96–4.63)	5 (5)	6.63 (2.38–18.48)	2.37 (0.63–8.89)	36 (35)	2.39 (1.54–3.72)	2.45 (1.54–3.87)
**Perineural growth**									
Absent	64 (31)	1.00	1.00	42 (17)	1.00	1.00	22 (14)	1.00	1.00
Present	103 (81)	2.65 (1.74–4.05)	1.03 (0.60–1.77)	19 (13)	2.10 (1.04–4.24)	2.37 (0.98–5.72)	84 (68)	1.88 (1.05–3.38)	0.94 (0.49–1.81)
**Growth in peripancreatic fat**									
Absent	62 (28)	1.00	1.00	40 (14)	1.00	1.00	22 (14)	1.00	1.00
Present	105 (84)	3.03 (1.95–4.72)	1.93 (1.17–3.18)	21 (16)	3.02 (1.49–6.11)	0.88 (0.12–6.70)	84 (68)	1.80 (1.00–3.25)	1.22 (0.63–2.36)
**Adjuvant treatment**									
Absent	92 (63)	1.00	1.00	43 (24)	1.00	1.00	49 (39)	1.00	1.00
Present	75 (49)	1.02 (0.70–1.49)	0.67 (0.45–0.99)	18 (6)	0.56 (0.23–1.36)	0.23 (0.09–0.64)	57 (43)	0.90 (0.58–1.39)	0.67 (0.43–1.04)
**pIgR expression**									
High	39 (14)	1.00	1.00	23 (5)	1.00	1.00	16 (9)	1.00	1.00
Low	128 (98)	2.99 (1.71–5.25)	1.98 (1.10–3.57)	38 (25)	3.90 (1.49–10.21)	3.76 (1.27–11.11)	90 (73)	1.68 (0.84–3.37)	1.39 (0.66– 2.96)

**Table 4:** Unadjusted and adjusted hazard ratios for recurrence in the entire cohort, intestinal type and pancreatobiliary type tumours.

	Entire cohort			Intestinal type			Pancreatobiliary type		
		Unadjusted	Adjusted		Unadjusted	Adjusted		Unadjusted	Adjusted
	n(events)	HR(95%CI)	HR(95%CI)	n(events)	HR(95%CI)	HR(95%CI)	n(events)	HR(95%CI)	HR(95%CI)
**Age**									
Continuous	167 (117)	1.00 (0.98–1.02)	1.01 (0.98–1.04)	61 (29)	0.99 (0.96 (1.03)	1.06 (1.01–1.11)	106 (88)	0.98 (0.96–1.01)	0.99 (0.96–1.03)
**Gender**									
Female	84 (53)	1.00	1.00	34 (11)	1.00	1.00	50 (42)	1.00	1.00
Male	83 (64)	1.42 (0.98–2.05)	1.00 (0.65–1.51)	27 (18)	2.31 (1.08–4.94)	2.21 (0.95–5.14)	56 (46)	106 (0.69–1.61)	0.78 (0.49–1.24)
**Tumour origin**									
Duodenum	13 (4)	1.00	1.00	13 (4)	1.00	1.00			
Ampulla-Intestinal type	48 (25)	2.16 (0.75–6.20)	2.63 (0.90–7.66)	48 (25)	2.18 (0.76–6.27)	2.71 (0.56–12.97)			
Ampulla-Pancreatobiliary type	19 (16)	4.67 (1.56–14.03)	1.85 (0.57–5.97)				19 (16)	1.00	1.00
Distal Bile duct	44 (38)	5.27 (1.88–14.80)	3.60 (1.23–10.53)				44 (38)	1.10 (0.61–1.98)	1.58 (0.81–3.05)
Pancreas	43 (34)	5.04 (1.78–14.25)	2.98 (1.00–8.83)				43 (34)	1.06 (0.58–1.93)	1.05 (0.56–1.99)
**Tumour size**									
Continuous	167 (117)	1.02 (1.00–1.03)	1.00 (0.98–1.02)	61 (29)	1.00 (0.97–1.02)	1.01 (0.96–1.07)	106 (88)	1.03 (1.01–1.05)	1.01 (0.99–1.04)
**T-stage**									
T1	6 (2)	1.00	1.00	4 (1)	1.00	1.00	2 (1)	1.00	1.00
T2	20 (9)	1.59 (0.34–7.37)	1.06 (0.22–5.09)	10 (3)	1.28 (0.13–12.30)	3.86 (0.37–40.01)	10 (6)	1.61 (0.19–13.36)	0.69 (0.07–6.38)
T3	102 (75)	4.42(1.08–18.05)	1.92 (0.44–8.40)	25 (9)	1.86 (0.24–14.71)	5.87 (0.64–53.58)	77 (66)	4.66 (0.64–33.96)	1.30 (0.16–10.59)
T4	39 (31)	4.87 (1.16–20.42)	2.67 (0.52–13.76)	22 (16)	5.44 (0.72–41.21)	17.90 (1.83–175.49)	17 (15)	4.31 (0.56–33.10)	2.34 (0.11–49.78)
**N-stage**									
N0	62 (32)	1.00	1.00	33 (11)	1.00	1.00	29 (21)	1.00	1.00
N1 (metastasis in 1-3 lgl)	64 (48)	2.33 (1.48–3.66)	1.91 (1.17–3.13)	19 (11)	2.07 (0.89–4.78)	1.00 (0.38–2.60)	45 (37)	2.17 (1.25–3.78)	2.06 (1.13–3.75)
N2 (metastasis in 4 or more lgl)	41 (37)	3.84 (2.366–6.25)	2.43 (1.40–4.21)	9 (7)	4.05 (1.55–10.59)	4.03 (1.23–13.13)	32 (30)	3.11 (1.72–5.61)	2.23 (1.18–4.21)
**Differentiation grade**									
Well-moderate	69 (40)	1.00	1.00	30 (11)	1.00	1.00	39 (29)	1.00	1.00
Poor	98 (77)	2.22 (1.51–3.27)	1.78 (1.18–2.71)	31 (18)	2.15 (1.02–4.57)	1.34 (0.39–4.58)	67 (59)	2.32 (1.45–3.71)	2.31 (1.39–3.85)
**Involved margins, status**									
R0	23 (7)	1.00	1.00	17 (3)	1.00	1.00	6 (4)	1.00	1.00
R1 & Rx	144 (110)	4.26 (1.98–9.16)	2.30 (1.04–5.07)	44 (26)	4.51 (1.36–14.94)	1.75 (0.45–6.73)	100 (84)	2.31 (0.84–6.36)	2.28 (0.81–6.45)
**Lymphatic growth**									
Absent	60 (28)	1.00	1.00	28 (5)	1.00	1.00	32 (23)	1.00	1.00
Present	107 (89)	2.77 (1.80–4.26)	1.30 (0.79–2.15)	33 (24)	6.16 (2.34–16.19)	11.19 (3.20–39.12)	74 (65)	1.77 (1.09–2.88)	1.10 (0.62–1.95)
**Vascular growth**									
Absent	126 (79)	1.00	1.00	56 (24)	1.00	1.00	70 (55)	1.00	1.00
Present	41 (38)	3.43 (2.28–5.15)	2.56 (1.64–4.02)	5 (5)	8.16 (2.85–23.30)	3.40 (1.05–11.08)	36 (33)	2.30 (1.47–3.61)	1.98 (1.23–3.20)
**Perineural growth**									
Absent	64 (29)	1.00	1.00	42 (15)	1.00	1.00	22 (14)	1.00	1.00
Present	103 (88)	3.49 (2.26–5.39)	1.09 (0.64–1.85)	19 (14)	2.72 (1.31–5.65)	0.88 (0.23–3.32)	84 (74)	2.93 (1.57–5.45)	1.85 (0.96–3.56)
**Growth in peripancreatic fat**									
Absent	62 (25)	1.00	1.00	40 (12)	1.00	1.00	22 (13)	1.00	1.00
Present	105 (92)	4.30 (2.71–6.80)	2.47 (1.45–4.22)	21 (17)	4.74 (2.23–10.10)	2.05 (0.26–16.24)	84 (75)	2.60 (1.42–4.75)	1.68 (0.89–3.19)
**Adjuvant treatment**									
Absent	92 (61)	1.00	1.00	43 (21)	1.00	1.00	49 (40)	1.00	1.00
Present	75 (56)	1.24 (0.86–1.78)	0.65 (0.43–0.97)	18 (8)	0.87 (0.38–1.96)	0.43 (0.16–1.17)	57 (48)	1.08 (0.70–1.65)	0.79 (0.48–1.28)
**pIgR expression**									
High	39 (15)	1.00	1.00	23 (5)	1.00	1.00	16 (10)	1.00	1.00
Low	128 (102)	2.89 (1.67–4.98)	1.31 (0.70–2.45)	38 (24)	4.01 (1.53–10.54)	1.46 (0.43–5.03)	90 (78)	1.45 (0.75–2.81)	0.86 (0.40–1.82)

The prognostic value of pIgR expression was also assessed using the continuous score (Table 5), whereby a significantly reduced HR in both unadjusted and adjusted analysis was demonstrated for increased pIgR expression for both death and recurrence within 5 years, in the entire cohort as well as in intestinal type tumours. A borderline significant association of increasing pIgR expression and an improved survival was seen for pancreatobiliary type tumours.

**Table 5:** Five-year overall and recurrence free survival according to continuous pIgR expression in the entire cohort, intestinal and pancreatobiliary type tumours.

	5-year overall survival		Recurrence free survival	
	Unadjusted	Adjusted	Unadjusted	Adjusted
	HR(95%CI)	HR(95%CI)	HR(95%CI)	HR(95%CI)
**Entire cohort (n = 167)**	0.64(0.52–0.79)	0.71 (0.56–0.89)	0.65 (0.53–0.80)	0.71 (0.57–0.90)
**Intestinal type (n = 61)**	0.58 (0.40–0.82)	0.49 (0.32–0.74)	0.58 (0.41–0.83)	0.59 (0.38–0.92)
**Pancreatobiliary type (n = 106)**	0.76 (0.57–1.01)	0.75 (0.55–1.03)	0.78 (0.59–1.03)	0.86 (0.61–1.22)

The prognostic value of pIgR expression was also examined in strata according to adjuvant chemotherapy; i.e. any vs none, gemcitabine vs other, or 5-FU analogue vs other, whereby no modifying effect of any form of treatment could be demonstrated (data not shown).

## Discussion

This is, to the best of our knowledge, the first study on the prognostic value of pIgR expression in pancreatic and periampullary adenocarcinoma. The results demonstrate that patients with low pIgR expression have adverse clinicopathological characteristics, and a significantly shorter RFS and OS.

In the study group, encompassing a comparatively large consecutive series of 175 patients surgically treated with pancreaticoduodenectomy, all having distant metastasis-free disease, pIgR expression was not predictive of response to any type of adjuvant chemotherapy, but the prognostic value of pIgR remained significant after adjustment for established clinicopathological characteristics, including morphology, i.e. pancreatobiliary and intestinal type, and adjuvant treatment. Given the heterogeneity of these tumours, we also performed survival analyses stratified by morphological type. The results demonstrated that the prognostic value of pIgR was significant in intestinal type tumours but not in pancreatobiliary type tumours. However, a similar trend was seen in the latter, in particular when the prognostic value of pIgR was assessed as a continuous variable. Moreover, the significant down-regulation of pIgR in lymph node metastases compared to primary tumours in the entire cohort, was more evident in pancreatobiliary type tumours than in intestinal type tumours, which supports a tumour suppressive role for pIgR in this type of tumours as well. The lowest expression of pIgR was seen in tumours of pancreatic origin, i.e. pancreatobiliary type and the highest in primary tumours of duodenal origin, i.e. intestinal type, which is in line with the more favourable prognosis in the latter [5], [6]. pIgR appears to be strongly expressed in normal mucosa of the gastrointestinal tract, including the duodenum, but also in normal pancreatic ductal but not acinar cells. The association of pIgR with intestinal differentiation in epithelial neoplasms is further supported by findings from our previous study on esophageal and gastric cancer, where pIgR expression was found to be significantly higher in both Barrett's esophagus and gastric intestinal metaplasia compared with normal squamous epithelium and gastric mucosa [17]. Of note, while high pIgR expression was found to be an independent factor for prolonged survival, its expression did not differ significantly between adenocarcinomas arising in a background with or without intestinal metaplasia [17].

The finding that a high pIgR expression is associated with more favourable clinicopathological characteristics and loss thereof with an adverse clinical outcome is in line with the vast majority of hitherto published studies in other cancer forms, e.g. gastro-esophageal [12], [17], ovarian [16], bladder [14], colon [11], and non-small cell lung cancer [13]. In this context, the correlation between protein and mRNA levels of PIGR seems to be good and decreased levels of both in malignant as compared with benign tissue has been observed in previous studies on e.g. lung and colorectal cancer [13], [22], [23]. The mechanistic basis underlying the potential tumour-suppressing role for pIgR in pancreatic and periampullary adenocarcinoma, as well as in several other cancer forms, remains to be elucidated. Given its important immunoregulatory function, the interplay between pIgR and the inflammatory microenvironment of tumours warrants further study. For instance, pIgR has been demonstrated to be downregulated in the intestinal mucosa in patients with inflammatory bowel disease, and levels of pIgR expression were found to correlate with the severity of the disease [24].

Of note, contrasting results, supporting a tumour-promoting role for pIgR, have been described in a comprehensive translational study on hepatocellular carcinoma (HCC), where high pIgR expression was found to be associated with early recurrence and chronic hepatitis B-virus (HBV)-infection [18]. Moreover, pIgR was found to induce epithelial-mesenchymal transition (EMT) *in vitro* and *in vivo* through activation of Smad signaling, suggesting a role for pIgR as a mediator of inflammation-induced EMT [18]. pIgR expression is frequently increased in response to viral or bacterial infections [8], [25], and HBV antigen-induced hepatocyte damage, followed by regeneration of hepatocytes, fibrosis and ultimately cirrhosis, are important events in hepatocellular carcinogenesis [26]. Therefore, the findings by Ai et al. indicate a link between HBV-related chronic inflammation and HCC metastasis [18]. Along this line, given the fact that an extensive desmoplastic stromal reaction is one of the hallmarks of pancreatic cancer [27], it may be hypothesized that elevated pIgR expression exerts tumour-promoting effects also in pancreatic and periampullary adenocarcinoma. In further support of this notion, Kadaba et al. indeed demonstrated a reciprocal relationship between expression of E-cadherin and pIgR in pancreatic cancer cells, and that this relationship, in turn, is dependent on the stromal content, in particular the proportion of activated stellate cells [19]. The reciprocal relationship between pIgR and E-cadherin was also confirmed in an analysis of 51 human ductal pancreatic cancer samples (TMA), further indicating a link between pIgR and EMT also in pancreatic cancer, but the associations with pIgR expression and clinical outcome were not described [19]. Thus, in the light of these studies on HCC and pancreatic cancer, the results from our study may seem contradicting and not in line with the expected. However, since our study cohort encompassed a larger cohort including pancreatic as well as periampullary adenocarcinoma, our findings further mirror the complexity and heterogeneous nature of tumours arising in the pancreas and periampullary region. It is therefore reasonable to assume that the role of pIgR in carcinogenesis and tumour progression may well differ by histological type and tumour origin, which should be considered in future translational studies on pancreatic and periampullary adenocarcinoma.

Given the important role of pIgR in ensuring the basal to apical transport and secretion of immunoglobulins in a variety of epithelial cells, another interesting avenue of research may be to explore the role of pIgR in the context of the regulation and function of cancer-associated immunoglobulins. It has recently been discovered that a variety of normal non-B and malignant cells also produce immunoglobulins, and accumulated experimental evidence indicates that these atypical immunoglobulins promote growth and proliferation of cancer cells [28], [29]. Cancer-associated immunoglobulin G has also been shown to enhance the growth and proliferation of cancer cells via induction of reactive oxygen species (ROS) [30]. Speculatively, pIgR may interact and modulate levels of cancer-cell derived immunoglobulins in certain malignancies, or subgroups thereof, thereby exerting either promoting or suppressive effects on carcinogenesis.

Some technical aspects and potential limitations to the present study should be noted. More than fifteen years after its introduction [31], the TMA technique can be considered a well-established platform for tissue biomarker studies, providing similar or even better prognostic information than full-face tissue section based analyses [32]. However, issues related to suboptimal sampling, e.g. of heterogeneously expressed markers, may still arise. To account for this, in the construction of the TMA used in the present study, tissue cores were, whenever possible, obtained from different donor blocks from the primary tumours, and from different lymph node metastases in cases with more than one metastasis. Moreover, the use of three 1.0 mm cores can be considered a comparatively generous sampling size and, of note, heterogeneity issues cannot be fully circumvented even by the use of full-face sections.

Another potential limitation is that the use of CRT analysis to determine the optimal prognostic cut-off for pIgR expression may lead to overfitting of the model. Therefore, the cut-off value applied in the present study should be evaluated in additional studies on tumour samples from independent patient cohorts. Analyses of the association of continuous pIgR expression with survival were however confirmatory, and, in the case of pancreatobiliary type tumours, an even stronger, although only borderline significant, association between high pIgR expression and a prolonged survival was observed.

## Conclusions

These findings demonstrate a variable expression of pIgR in pancreatic and periampullary adenocarcinoma, with low versus high expression being significantly associated with several adverse tumour characteristics, progressive disease and a shorter time to recurrence and death within five years. The mechanistic basis for the role of pIgR in the carcinogenesis and progression of these heterogeneous cancers, with the common denominator of having a dismal prognosis, merits further study.

## Supporting Information

Figure S1 **PIGR mRNA levels in paired primary tumours and lymph node metastases from two cases.** Real-time quantitative PCR analysis of PIGR mRNA levels in A) an intestinal type tumour with high pIgR expression in both the primary tumour and metastasis and B) in a pancreatobiliary type tumour with intermediate pIgR expression in the primary tumour and negative expression in the metastasis. NS =  Nuclear score.
(TIF)

Figure S2 **Classification and regression tree analysis for the selection of prognostic cut-off for 5-year overall survival in the entire cohort.**
(DOCX)
