# Supplementary figures and images for: Reduced Expression of the Polymeric Immunoglobulin Receptor in Pancreatic and Periampullary Adenocarcinoma Signifies Tumour Progression and Poor Prognosis
Source: PLoS One. 2014 Nov 14;9(11):e112728. doi: 10.1371/journal.pone.0112728 (PMC4232506; doi:10.1371/journal.pone.0112728)

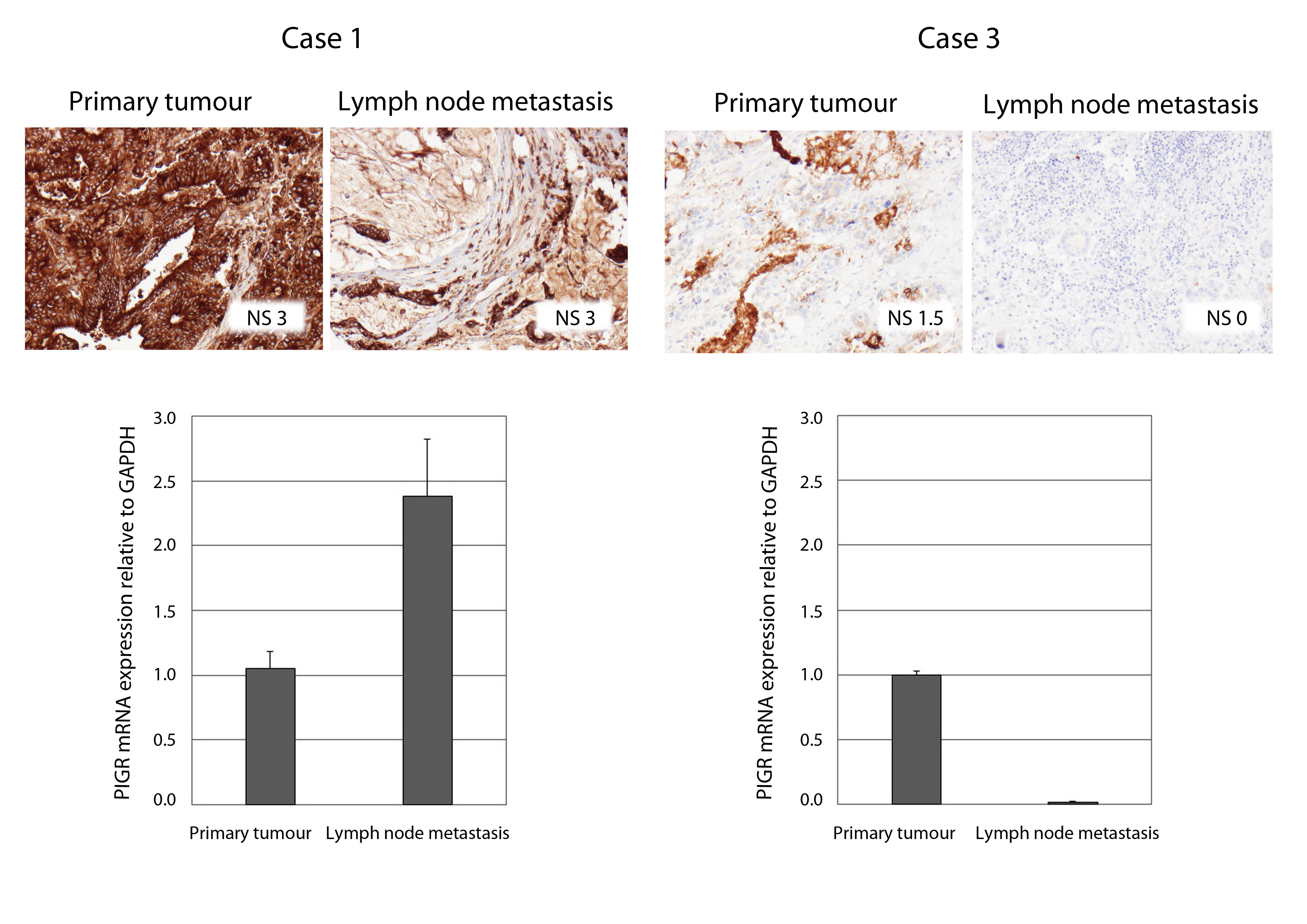

Supplement: Figure S1 — PIGR mRNA levels in paired primary tumours and lymph node metastases from two cases. Real-time quantitative PCR analysis of PIGR mRNA levels in A) an intestinal type tumour with high pIgR expression in both the primary tumour and metastasis and B) in a pancreatobiliary type tumour with intermediate pIgR expression in the primary tumour and negative expression in the metastasis. NS = Nuclear score. (TIF) [file pone.0112728.s001.tif]

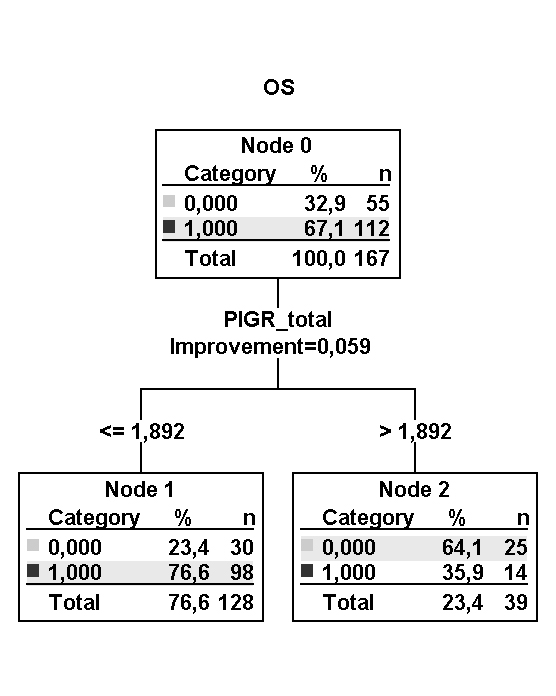

Supplement: Figure S2 — Classification and regression tree analysis for the selection of prognostic cut-off for 5-year overall survival in the entire cohort. (DOCX) [file pone.0112728.s002.docx]
